# Deep Learning-Based Diffusion-Weighted Magnetic Resonance Imaging in the Diagnosis of Ischemic Penumbra in Early Cerebral Infarction

**DOI:** 10.1155/2022/6270700

**Published:** 2022-02-28

**Authors:** Hui Sheng, Xueling Wang, Meiping Jiang, Zhongsheng Zhang

**Affiliations:** ^1^Department of Radiology, Yantaishan Hospital, Yantai 264000, China; ^2^Department of Ultrasonic, Yuhuangding Hospital, Yantai 264000, China; ^3^Department of Radiology, Yuhuangding Hospital, Yantai 264000, China

## Abstract

The prefiltered image was imported into the local higher-order singular value decomposition (HOSVD) denoising algorithm (GL-HOSVD)-optimized diffusion-weighted imaging (DWI) image, which was compared with the deviation correction nonlocal mean (NL mean) and low-level edge algorithm (LR + edge). Regarding the peak signal-to-noise ratio (PSNR), root mean square error (RMSE), sensitivity, specificity, accuracy, and consistency, the application effect of the GL-HOSVD algorithm in DWI was investigated, and its adoption effect in the examination of ischemic penumbra (IP) of early acute cerebral infarction (ACI) patients was evaluated. A total of 210 patients with ACI were selected as the research subjects, who were randomly rolled into two groups. Those who were checked by conventional DWI were set as the control group, and those who used DWI based on the GL-HOSVD denoising algorithm were set as the observation group, with 105 people in each. Positron emission tomography (PET) test results were set as the gold standard to evaluate the application value of the two examination methods. It was found that under different noise levels, the peak signal-to-noise ratio (PSNR) of the GL-HOSVD algorithm and the root mean square error (RMSE) of the FA parameter were better than those of the nonlocal means (NL-means) of deviation correction and low-rank edge algorithm (LR + edge). The sensitivity, specificity, accuracy, and consistency (8.76%, 81.25%, 87.62%, and 0.52) of the observation group were higher than those of the control group (57.78%, 53.33%, 57.14%, and 0.35) (*P* < 0.05). Moreover, the apparent diffusion coefficient (ADC) of the DWI images of the observation group was basically consistent with that of the PET images, while the control group had a poor display effect and low definition. In summary, under different noise levels, the GL-HOSVD algorithm had a good denoising effect and greatly reduced fringe artifacts. DWI after denoising had high sensitivity, specificity, accuracy, and consistency in the detection of IP, which was worthy of clinical application and promotion.

## 1. Introduction 

The incidence rate of acute cerebral infarction (ACI) is high, and there is a high rate of disability and mortality, which poses a great threat to human life, especially to the middle-aged and elderly [1, 2]. Studies suggested that an ischemic penumbra (IP) exists in abnormal brain tissue in ACI patients [3]. IP refers to the hypoperfusion of the ischemic tissue surrounding the center of cerebral infarction lesions. Although there is a possibility of loss of neural electrical movement, the tissue structure is basically intact and the ionization balance is relatively normal [4]. The area of the IP changes dynamically and will decrease with the prolongation of ischemia time, but the infarct area will increase, and IP can be treated by blood flow recanalization [5]. Clinical studies found that the IP plays a key guiding role in the treatment of early cerebral infarction [6]. The accurate diagnosis of the IP is the vane for the rational implementation of intra-arterial thrombolytic therapy in early cerebral infarction patients.

At present, diffusion-weighted imaging (DWI) is commonly used in clinical diagnosis of cerebral ischemic lesions [7]. DWI is very sensitive to the diffusion of water molecules in tissues. The apparent diffusion coefficient (ADC) is the embodiment of the diffusion ability of water molecules in tissues. DWI uses the grayscale signal of the image to reflect the motion of water molecules, including the direction and degree of motion limitation, so as to indirectly reflect the microstructure of tissues and provide useful information for clinical diagnosis [8]. However, high noise exists in DWI images, especially in high-resolution or high B-value imaging [9], which will make image details blurred and affect the clinical diagnosis analysis. Therefore, the deep learning algorithm is applied to image denoising of DWI [10]. MR image denoising algorithm based on higher-order singular value decomposition (HOSVD) [11] has a good denoising effect, especially in T1-weighted (T1w), T2-weighted (T2w), and proton density-weighted (PDw) image noise processing [12–14]. However, compared with conventional MR, there is more redundant information in DWI images. In DWI image processing, the HOSVD algorithm can remove noise well and retain details, but there will be artifacts in the local uniform area. Some people proposed that global HOSVD denoising can be used to prefilter DWI images, and then the prefiltered images can be used to guide the subsequent local denoising; this method was referred to as GL-HOSVD.

Therefore, in this study, DWI images based on the GL-HOSVD denoising algorithm were used to examine the IP of patients with early ACI, and the accuracy of the examination results was evaluated. It aimed to provide more effective examination methods for patients with early cerebral infarction and provide more possibilities for early diagnosis and treatment of patients.

## 2. Methods

### 2.1. Research Objects

In this study, 210 patients diagnosed with ACI admitted to the hospital from January 2018 to January 2021 were selected as the research subjects. There were 145 male patients and 65 female patients, ranging in age from 35 to 78 years, with an average age of (60.17 ± 7.98) years. The course of the disease ranged from 3 to 6 hours, with an average course of (4.17 ± 1.52) hours. The patients were randomly divided into two groups (the control and observation groups), with 105 patients in each group. The control group was given a brain scan using conventional DWI. The group of patients who were examined and diagnosed by DWI based on the GL-HOSVD denoising algorithm was set as the observation group. The results of positron emission tomography (PET) [15, 16] were used as the gold standard to evaluate the application value of the two tests. This study has been approved by the medical ethics committee of the hospital, and all patients and their families signed informed consent.

Inclusion criteria were defined as follows: (i) all patients had cerebral cortical infarction; (ii) the National Institute of Health Stroke Scale (NIHSS) score ≥4 at ACI onset; (iii) the onset time of the patient was less than 24 hours; (iv) thrombolytic therapy was performed before; and (v) patients and their families had been informed and consented. Exclusion criteria were defined as follows: (i) patients with serious heart, liver, and kidney dysfunction, cancer, malnutrition, and autoimmune diseases; (ii) patients with previous stroke; (iii) patients with cerebral hemorrhage; (iv) patients with adverse reactions to DWI examination; and (v) patients with unclear images or incomplete sequences in the DWI examination.

### 2.2. Denoising Algorithm Based on GL-HOSVD

**Figure 1:**
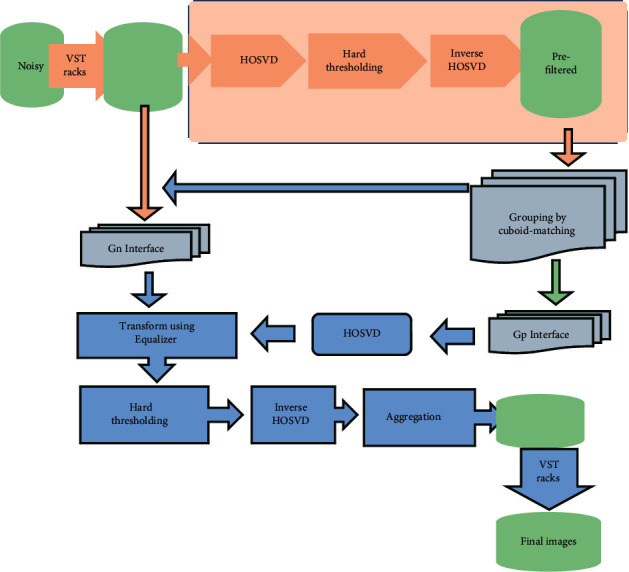
GL-HOSVD algorithm denoising process.

GL-HOSVD denoises the DWI image in two steps. The first step is global HOSVD denoising, and the two-dimensional DWI image in each diffusion direction is regarded as a third-order tensor. The HOSVD algorithm is used to transform and hard threshold this third-order tensor, and the prefiltered image is obtained through the inverse transformation of HOSVD. The second step is local HOSVD denoising, combining two-dimensional and three-dimensional similar blocks to turn them into high-order tensors. HOSVD inverse transform is performed to denoise, and the similar block group after denoising is obtained. Then, the multiple estimated values of each pixel are weighted and averaged to get the final denoised image, as explained in Figure 1.

#### 2.2.1. Global HOSVD Denoising

It is assumed that the DWI noise image has *Q* diffusion coding directions; then, the third-order tensor composed of them can be expressed as the following equation:

(1) $$\begin{array}{c} Y\in {\mathbb{Z}}^{H\times W\times Q}, \end{array}$$

where *H* represents the height of the DWI image, *W* represents the width of the DWI image, and the noise-free image represented by *X* ∈ *ℤ*^*H*×*W*×*Q*^ and *Y* ∈ *ℤ*^*H*×*W*×*Q*^ is formed into a third-order tensor; the HOSVD decomposition algorithm of *Y* is as the following equation:

(2) $$\begin{array}{c} Y=S{\;\times \;}_{1}O^{\left(1\right)}{\;\times \;}_{2}O^{\left(2\right)}{\;\times \;}_{3}O^{\left(3\right)}, \end{array}$$

where *S* represents the core tensor, *S* ∈ *ℤ*^*H*×*W*×*Q*^, *O* represents the orthogonal identity matrix, *O*^(1)^ ∈ *ℤ*^*H*×*H*^, *O*^(2)^ ∈ *ℤ*^*W*×*W*^, *O*^(3)^ ∈ *ℤ*^*Q*×*Q*^, and *x*_*n*_ represents the *n*-modulus product of the tensor and the matrix. *O*^(*n*)^, *n*=1,2,3 represents the left singular vector obtained by decomposing the tensor *Y*.

To reduce the noise, the algorithm zeroes the transform coefficients from the noise in the tensor *S*, and the specific expression is as follows:

(3) $$\begin{array}{c} H_{\tau_{\mathrm{global}}}\left(S\right)=\left\{\begin{array}{cc} s_{i_{1}i_{2}i_{3}}, & \mathrm{if}\;abs\left(s_{i_{1}i_{2}i_{3}}\right)\geq \tau ,\\ 0, & \mathrm{if}\;abs\left(s_{i_{1}i_{2}i_{3}}\right)<\tau , \end{array}\right. \end{array}$$

where *H*_*τ*_global__ represents the hard threshold operation of *τ*_global_ and *s*_*i*_1_*i*_2_*i*_3__ represents the coefficient of the position (*i*_1_, *i*_2_, *i*_3_) in the tensor *S*. The threshold *τ*_global_ can be expressed as the following equation:

(4) $$\begin{array}{c} \tau_{\mathrm{global}}⟶q_{\mathrm{global}}\sigma \sqrt{2\;\log\left(H\times W\times Q\right)}, \end{array}$$

where *q*_global_ > 0 represents a scalar, which controls the smoothness in the denoising step, and *σ* represents the noise variance.

It is assumed that the coefficient tensor after the hard threshold operation is $\bar{S}$; then, there is the following equation:

(5) $$\begin{array}{c} \bar{S}=H_{\tau }\left(S\right). \end{array}$$

When the HOSVD base and the truncated tensor $\bar{S}$ are known, the denoised image obtained by the inverse HOSVD transformation can be expressed as the following equation:

(6) $$\begin{array}{c} \bar{X}=\bar{S}{\;\times \;}_{1}O^{\left(1\right)}{\;\times \;}_{2}O^{\left(2\right)}{\;\times \;}_{3}O^{\left(3\right)}, \end{array}$$

where $\bar{X}$ is expressed as the estimated value of *X*, which is the estimated value of the denoised image.

#### 2.2.2. Local HOSVD Denoising

*m* × *m* × *Q* represents the size of the three-dimensional block; then, the size of the spatial domain is *m* × *m*, and the size of the third three-dimensional is *Q*, i.e., the number of diffusion directions. To reduce the influence of noise on block grouping, the global prefiltered image obtained in the first step is used to calculate the grayscale distance between the two blocks, which is specifically expressed as the following equation:

(7) $$\begin{array}{c} d\left(P_{i},P_{j}\right)=\frac{{\left\|P_{i}-P_{j}\right\|}_{2}^{2}}{m^{2}Q}, \end{array}$$

where *P*_*i*_ represents a three-dimensional block centered on pixel *i*, *P*_*j*_ represents a three-dimensional block centered on pixel *j*, and *m*^2^*Q* represents the number of pixels in the three-dimensional block. Then, a block similar to the reference block is the following equation:

(8) $$\begin{array}{c} S_{i}^{th}=\left\{j\in X_{i}:d\left(P_{i},P_{j}\right)\leq \tau_{d}\right\}. \end{array}$$

In the above equation, *S*_*i*_^*th*^ represents the block coordinate set similar to the reference block *P*_*i*_, *X*_*i*_ represents the search window centered on pixel *i*, and *τ*_*d*_ represents the threshold value on the distance. The total number of similar blocks is *L*. Under circumstance that *S*_*i*_^*th*^ is known, two similar block groups are constructed, which are derived from the original noise image (denoted as *G*_*n*_ ∈ *ℤ*^*m*×*m*×*Q*×*L*^) and the prefiltered image (denoted as *G*_*p*_ ∈ *ℤ*^*m*×*m*×*Q*×*L*^). If the latter is less affected by noise than the former, the obtained HOSVD basis *G*_*p*_ ∈ *ℤ*^*m*×*m*×*Q*×*L*^ is transformed into the former, which can be expressed as the following equation:

(9) $$\begin{array}{c} G_{p}=S_{p}{\;\times \;}_{1}O_{p}^{\left(1\right)}{\;\times \;}_{2}O_{p}^{\left(2\right)}{\;\times \;}_{3}O_{p}^{\left(3\right)}{\;\times \;}_{4}O_{p}^{\left(4\right)}, \end{array}$$

where *S*_*p*_ represents the fourth-order core tensor, *S*_*p*_=*ℤ*^*m*×*m*×*Q*×^, *O*_*p*_^(*n*)^ represents the orthogonal identity matrix, and *O*_*p*_^(1)^, *O*_*p*_^(1)^ ∈ *ℤ*^*m*×*m*^, *O*_*p*_^(3)^ ∈ *ℤ*^*Q*×*Q*^, and *O*_*p*_^(4)^ ∈ *ℤ*^*L*×*L*^. The following equation can be obtained by importing *G*_*n*_ ∈ *ℤ*^*m*×*m*×*Q*×*L*^ into *O*_*p*_^(1)^, *O*_*p*_^(2)^, *O*_*p*_^(3)^, *O*_*p*_^(4)^.

(10) $$\begin{array}{c} S_{n}=G_{n}{\;\times \;}_{1}O_{p}^{{\left(1\right)}^{T}}{\;\times \;}_{2}O_{p}^{{\left(2\right)}^{T}}{\;\times \;}_{3}O_{p}^{{\left(3\right)}^{T}}{\;\times \;}_{4}O_{p}^{{\left(4\right)}^{T}}. \end{array}$$

Among them, *S*_*n*_ represents the fourth-order core tensor transformed by *O*_*p*_^(1)^, *O*_*p*_^(2)^, *O*_*p*_^(3)^, *O*_*p*_^(4)^, and *S*_*n*_ ∈ *ℤ*^*m*×*m*×*Q*×*L*^. After that, a hard threshold operation is performed on *S*_*n*_. It is set that $\tau_{\mathrm{global}}⟶q_{\mathrm{global}}\sigma \sqrt{2\;\log\left(m\times m\times Q\times L\right)}$, *k*_global_ > 0 denotes a scalar, and the smoothness in the denoising step is controlled. The truncation coefficient after the hard threshold operation of *S*_*n*_ is denoted as $\bar{S_{n}}\in {\mathbb{Z}}^{m\times m\times Q\times L}$; the corresponding similar block $\bar{G_{n}}$ after denoising is the following equation:

(11) $$\begin{array}{c} \bar{G_{n}}=\bar{S_{n}}{\;\times \;}_{1}O_{p}^{\left(1\right)}{\;\times \;}_{2}O_{p}^{\left(2\right)}{\;\times \;}_{3}O_{p}^{\left(3\right)}{\;\times \;}_{4}O_{p}^{\left(4\right)}. \end{array}$$

The above equations (7)–(11) are operated in the form of a sliding window on the image as a whole, and *N*_step_ is used to represent the sliding step length. The smaller the *N*_step_, the better the denoising effect, but it will reduce the calculation efficiency. To solve this problem, a weighted average operation is performed on multiple estimated values of pixels, as follows:

(12) $$\begin{array}{c} \bar{x_{i}}=\frac{\sum_{j=1}^{V}\theta_{j}\sum_{q=1}^{Q_{j}}{\bar{G}}_{j}^{q}\left(i\right)}{\sum_{j=1}^{V}Q_{j}\theta_{j}},\\ \theta_{j}=\frac{1}{1+{\left\|\bar{S_{j}}\right\|}_{0}}, \end{array}$$

where $\bar{x_{i}}$ represents the filter value of pixel *i*, ${\bar{G}}_{j}\left(i\right)$ represents the value of pixel *i* in the *q*-th block of similar blocks ${\bar{G}}_{j}$, *Q*_*j*_ represents the number of blocks of pixel *i*, *V* represents the number of ${\bar{G}}_{j}$, *θ*_*j*_ represents the weight of ${}_{\bar{G}}^{j}$, and ${\left\|\bar{S_{j}}\right\|}_{0}$ represents the *l*_0_-norm of $\bar{S_{j}}$.

#### 2.2.3. Evaluation Methods

The peak signal-to-noise ratio (PSNR) [17] and the root mean square error (RMSE) of FA parameters [18] are commonly used denoising performance evaluation standards. Specifically, they are expressed as the following equation:

(13) $$\begin{array}{c} \text{PSNR}=10\;\log_{10}\left(\frac{M}{{\left\|\bar{I}-I\right\|}_{2}^{2}}\right). \end{array}$$

In the equation, *I* represents the noise-free DWI image, $\bar{I}$ represents the image after denoising, and *M* represents the number of pixels in the image.

(14) $$\begin{array}{c} \mathrm{FA}-\mathrm{RMSE}=\sqrt{\frac{{\left\|{\bar{I}}_{\text{FA}}-I_{\text{FA}}\right\|}_{2}^{2}}{M_{\text{FA}}}}, \end{array}$$

where *I*_FA_ represents the FA image obtained by noise-free estimation, ${\bar{I}}_{\text{FA}}$ represents the FA image obtained by estimation after denoising, and *M*_FA_ represents the total number of pixels in the FA image.

### 2.3. Inspection Methods

#### 2.3.1. DWI Scan

3.0 T superconducting magnetic resonance imaging scanner was used which supports orthogonal coils for the head. During the scanning process, the patient's head was placed in the center of the coil, and the coronal scan was performed with the optic chiasm as the center. The echo planar imaging (EPI) was used, and the specific scanning parameters are shown in Table 1. After scanning to obtain the DWI image with the above parameters, the ADC image was obtained by reconstruction using the DWI image, and the ADC value was obtained. The ADC value is calculated according to the following equation:

(15) $$\begin{array}{c} \text{ADC}=\frac{I_{n}\left(S_{n}-S_{1}\right)}{b_{1}-b_{n}}, \end{array}$$

where *b* represents the dispersion sensitivity coefficient, *S*_1_ represents the signal strength in the case of *b*_1_, *S*_*n*_ represents the signal strength in the case of *b*_*n*_, and *I*_*n*_ represents the mathematical symbol.

#### 2.3.2. PET Scan

During the PET scan, the patient was placed prone on the PET examination table and was anesthetized using inhalation anesthesia. During the scanning process, the patient's head should be kept stable, the transmission and emission imaging should be performed according to the programmed procedures, and the tissue attenuation correction of the corresponding images should be collected. The imaging was reconstructed using the Maximum A Posteriori (MAP) method. The pixels of the PET image after imaging were 0.5 × 0.5 × 1.0 mm^3^, and the layer thickness was 1.20 mm. Then, the recovery of local brain glucose metabolism was observed.

### 2.4. Observation Indexes

Based on the results of PET examinations, the sensitivity (Sen), specificity (Sep), and accuracy (Acc) of the two inspection methods to IP detection after ACI were analyzed. The Kappa value of the consistency was compared between the two groups of examination results and the PET examination results. If Kappa=1, then the diagnosis results were exactly the same. If Kappa = −1, then the diagnosis results were completely inconsistent. If Kappa ≥ 0.75, then the degree of agreement in the diagnosis was quite satisfactory. If Kappa < 0.4, then the consistency of the diagnosis results was not ideal. If 0.75 > Kappa ≥ 0.4, then the diagnosis consistency was relatively satisfactory.

(16) $$\begin{array}{c} \text{Sen}=\frac{\mathrm{TP}}{\mathrm{TP+FN}},\\ \mathrm{Sep}=\frac{\mathrm{TN}}{\mathrm{TN+FP}},\\ \mathrm{Acc}=\frac{\mathrm{TP+TN}}{\mathrm{TP+TN+FP+FN}}. \end{array}$$

True positive (TP) refers to the number of correctly classified positive samples, i.e., the predicted positive samples are actually positive samples. False positive (FP) refers to the number of negative samples incorrectly marked as positive samples, i.e., the actual negative samples are predicted as positive samples. True negative (TN) refers to the number of correctly classified negative samples, i.e., the predicted negative samples are actually negative samples. False negative (FN) refers to the number of positive samples incorrectly marked as negative samples, i.e., the actual positive samples are predicted to be negative samples. TP + FP + TN + FN refers to the total number of samples; TP + FN refers to the actual positive sample number; TP + FP refers to the total number of positive samples, including correct and incorrect predictions. FP + TN refers to the actual negative sample number; TN + FN refers to the total number of samples with negative predicted results, including correct and wrong predictions.

### 2.5. Statistical Analysis

SPSS 22.0 was used for data entry, sorting, and statistical analysis. *χ*^2^ test was used to compare the count data. The *T* test was used to compare the measurement data. Analysis of variance was used to compare the mean values of multiple samples. The LSD method was used when the mean values were uniform, and the Dunnett T3 method was used when the mean values were uneven. *P* < 0.05 showed statistical difference.

## 3. Results

### 3.1. Comparison of Denoising Performance

**Figure 2:**
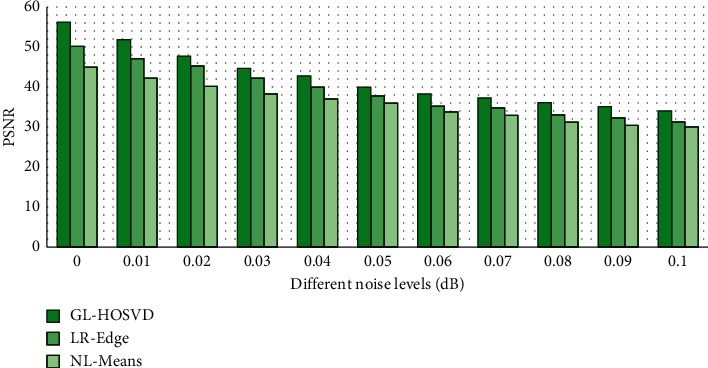
PSNR comparison results.

**Figure 3:**
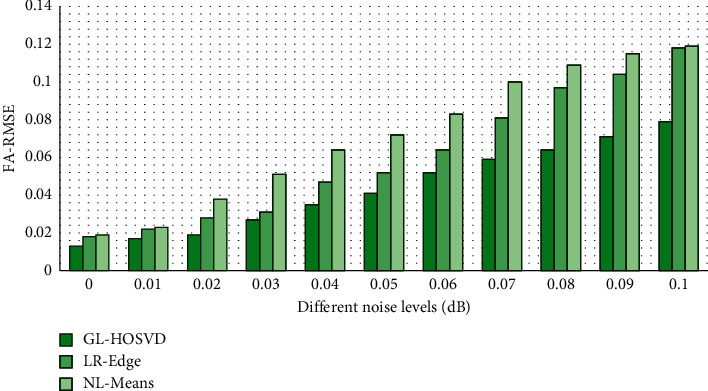
FA-RMSE.

**Figure 4:**
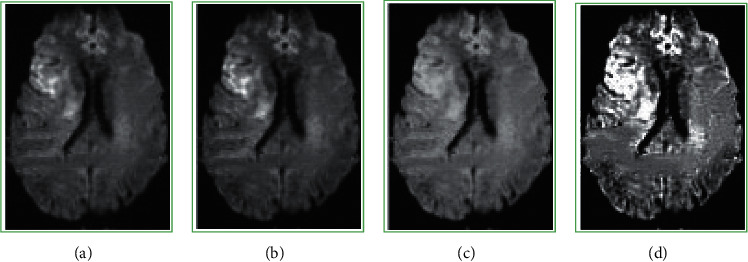
Comparison of treatment effects. (a) Original Image, (b) LR-Edge, (c) NL-Means, and (d) GL-HOSVD.

The GL-HOSVD algorithm, the deviation-corrected nonlocal means (NL-Means) [19] algorithm, and the low-rank edge preservation algorithm (LR + Edge) [20] were compared regarding the denoising effect under different noise levels (N). The value of the noise level C was 0 to 0.1.  Figure 2 compares the results of the PSNR, while Figure 3 compares the results of FA-RMSE. It was found that under different noise levels, the PSNR and FA-RMSE values of the GL-HOSVD algorithm were better than the NL-Means and LR + Edge algorithms.  Figure 4 shows a specific denoising processing effect diagram, and it was obvious that the image processed by the GL-HOSVD algorithm had higher definition and more prominent details.

### 3.2. General Information Comparison

**Figure 5:**
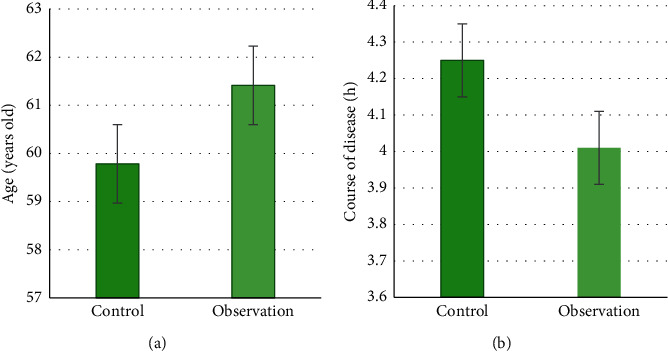
Baseline data. (a) The average age; (b) the average disease course.

According to the statistics of general treatment of patients, in terms of gender distribution, there were 71 male patients (48.97%) and 34 female patients (52.31%) in the control group. There were 74 male patients (51.03%) and 31 female patients (47.69%) in the observation group. There was no considerable difference in comparison (*P* > 0.05). In terms of age distribution, the average age of the patients in the control group was (59.78 ± 8.98) years, and the average age of the patients in the CT group was (61.41 ± 9.01) years, with no considerable difference (*P* > 0.05) (Figure 5(a)). In terms of the distribution of the disease course, the average course of the disease in the control group was (4.25 ± 1.61) hours, and the average course of the observation group was (4.01 ± 1.47) hours. There was no considerable difference (*P* > 0.05) (Figure 5(b)). The above comparisons indicated that the two groups in this study were comparable.

### 3.3. Examination Result Statistics

**Figure 6:**
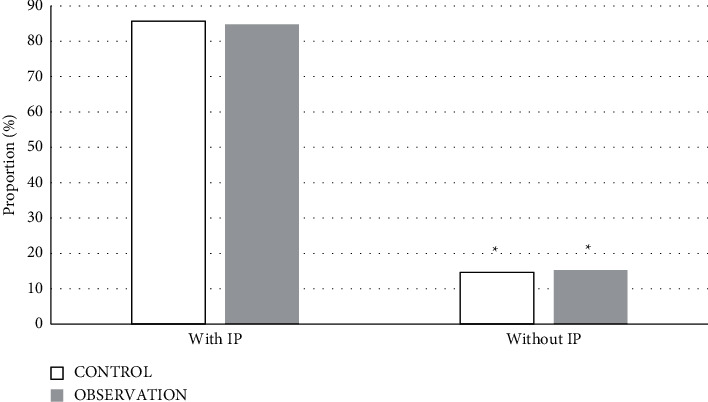
Comparison of the results of patients with IP and without IP (^*∗*^ represents comparison of the proportion of patients with IP, *P* < 0.05).

Tables 2 and 3 show the two inspection methods of the control group and the observation group, as well as the statistics of the IP results detected by the PET inspection. The inspection results were organized using a four-grid table. According to the test results of PET, most of the two groups of patients had IP. The proportion of patients with IP in the control group was 85.71%, and the proportion of patients without IP was 14.59%. In the observation group, the proportion of patients with IP was 84.76%, and the proportion of patients without IP was 15.24%. The comparison between the proportions within the group was statistically considerable (*P* < 0.05) (Figure 6).

### 3.4. Comparison of Sensitivity, Specificity, Accuracy, and Consistency

**Figure 7:**
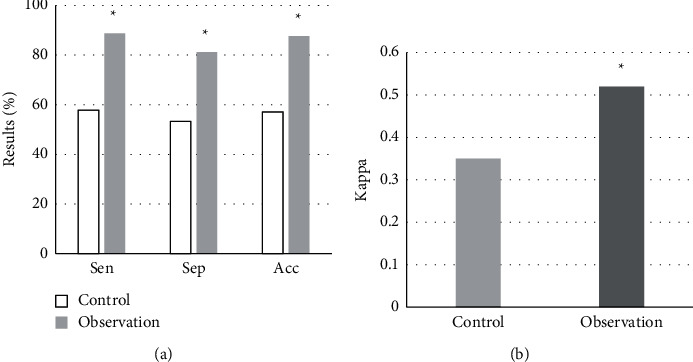
Comparison of detection effects. (a) Sensitivity, specificity, and accuracy; (b) consistency; ^*∗*^represents *P* < 0.05 compared with the control group.

According to Tables 2 and 3, the sensitivity, specificity, accuracy, and consistency of the two test methods were calculated and analyzed. The results showed that the sensitivity, specificity, and accuracy of the test method in the control group were 57.78%, 53.33%, and 57.14%, respectively, and the consistency Kappa value with the PET test results was 0.35. The sensitivity, specificity, and accuracy of the test methods in the observation group were 88.76%, 81.25%, and 87.62%, respectively, and the consistency Kappa value with the PET test results was 0.52. After comparative analysis, the sensitivity, specificity, accuracy, and Kappa of the observation group were better than those of the control group, and the differences were statistically considerable (*P* < 0.05) (Figure 7).

### 3.5. ADC Comparison

Table 4 shows the average ADC values of the infarct lesion core and the IP region of IP patients detected by DWI in the two groups. After comparative analysis, the ADC value of the infarct lesion core in the control and observation groups ((0.342 ± 0.106) × 10^−3^ mm^3^/s and (0.336 ± 0.102) × 10^−3^ mm^3^/s) was lower than the ADC value in the IP area of the two groups ((0.803 ± 0.149) × 10^−3^ mm^3^/s and (0.793 ± 0.136) × 10^−3^ mm^3^/s), and there were statistical differences in comparison (*P* < 0.05).

### 3.6. Examination Image Comparison

**Figure 8:**
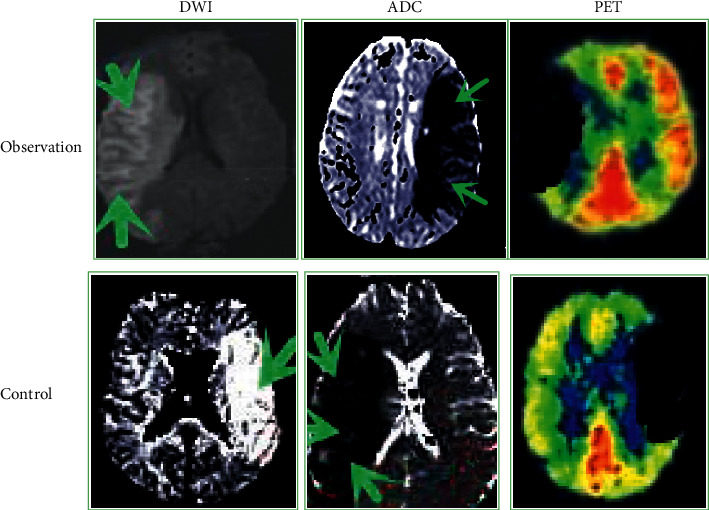
Image comparison (the green arrow indicates the focal area. Control group: a 65-year-old male with a 4-hour course of disease; observation group: a 63-year-old male with a 3.5-hour course of disease).

Figure 8 shows the comparison of DWI images, ADC images, and PET results of the two groups of patients with IP detected by conventional DWI and denoised DWI. Through observation, PET examination can highlight the IP region through the glucose metabolism in the brain, and the two sets of ADC images can also show abnormal signals in the IP region of the brain. The display results of the DWI images in the observation group were basically consistent with the corresponding ADC image results and clarity, while the DWI images in the control group were worse than their corresponding ADC images with lower clarity.

## 4. Discussion

The accurate diagnosis of IP is an important basis for thrombolytic therapy for patients with early cerebral infarction. Therefore, clinical studies on the diagnosis of IP have attracted wide attention [21]. In this study, DWI was used to examine IP in patients with ACI, and the value of the examination results was evaluated.

Firstly, the GL-HOSVD algorithm was employed to denoise the DWI images of the observation group, and the denoising performance was compared with other algorithms (NL-Means algorithm and LR + Edge algorithm). The results showed that under different noise levels, the PSNR and FA-RMSE values of the GL-HOSVD algorithm were better than those of the NL-Means and LR + Edge algorithm. Zhang et al. [22] also carried out experiments on denoising DWI data by introducing the global HOSVD algorithm into the local HOSVD algorithm. The results showed that the proposed method greatly reduced the fringe artifacts and was superior to the two latest denoising methods (NL-Means and LR + Edge algorithm) in terms of denoising quality and diffusion parameter estimation. Wu et al. [23] used the HOSVD algorithm super resolution (SR) technology to reconstruct DWI datasets with high discrimination rate and achieved good results, which reflected that the improved HOSVD algorithm had a good effect on DWI image denoising, which was consistent with the results of this work. The HOSVD algorithm has also been applied to other imaging technologies, such as PET [24], hyperpolarized 13C magnetic resonance image [25], and CT [26], all of which have achieved good research results.

Then, the sensitivity, specificity, and accuracy of DWI images denoised by the GL-HOSVD algorithm were compared with conventional DWI image detection results. The results of the observation group were 8.76%, 81.25%, and 87.62%, respectively, which were greatly higher than 57.78%, 53.33%, and 57.14% of the control group, and the comparison was statistically considerable (*P* < 0.05). In terms of consistency with PET test results, the Kappa value of the observation group was 0.52, suggesting that the consistency of the DWI diagnosis was relatively satisfactory. However, the Kappa value of the control group was 0.35, indicating poor consistency of diagnosis. The above comparison results suggested that the denoised DWI image had a more prominent effect on the display of the IP phenomenon, which was worthy of clinical promotion. The study suggested that conventional DWI detection alone could not effectively diagnose IP in patients with early cerebral infarction [27–29], which was consistent with the above comparison results. In the study, ADC, DWI, and PET images were compared. The results showed that the IP display effect of ADC and PET images in the two groups was basically similar to that of the denoised DWI images, indicating that the IP inspection effect of ADC images was superior to conventional DWI images. Zhang et al. [30] proposed that the radio metrics model based on the ADC graph can effectively determine whether IP exists in patients with early cerebral infarction. The results of Malik et al. [31] also suggested the prominent effect of ADC imaging in brain detection. The observation group's processed DWI images, ADC images, and PET images had basically the same inspection effect. It indicated that the DWI image denoised by the GL-HOSVD algorithm can be better applied to the IP inspection. In addition, it was found that the IP phenomenon existed in most of the two groups of ACI patients in this study, accounting for more than 80% of the total number of patients. These results indicated that the IP phenomenon had a relatively large probability in patients with ACI. However, there was a lack of correlation studies on this point, so there was no comparison with previous studies, leading to the underrepresentation of this result.

## 5. Conclusion

In this study, DWI images based on the GL-HOSVD denoising algorithm were used to examine the IP of patients with early ACI, and the accuracy of the examination results was evaluated. The results showed that the GL-HOSVD algorithm had a good denoising effect under different noise levels and greatly reduced fringe artifacts. Moreover, the denoised DWI had high sensitivity, specificity, accuracy, and consistency in IP detection, which was worthy of clinical application and promotion. However, this study did not compare the DWI inspection effect after denoising with other evaluation effects such as DWI-MTT mismatching area and DWI-SWI mismatching area, so the comparison lacked certain comprehensiveness and should be improved in the future. In conclusion, this study fully reflects that the deep learning algorithm has a good development prospect in the field of imaging, and its clinical auxiliary effect can be expected in the future.

## Figures and Tables

**Table 1:** Scanning parameters.

Index	Repeat time (ms)	Echo time (ms)	Layer thickness (mm)	Layer spacing (mm)	Matrix	Field	*b*	Flip angle	Number of incentives	Scan time (s)
Value	3647	107	6	1.5	192 × 192	22 cm × 22 cm	1000s/mm^2^	90°	Twice	78

**Table 2:** Statistics of the inspection results of the control group.

Control group (*n* = 105 cases)		PET		Total
		With IP	Without IP	
Conventional DWI	With IP	52	7	59
	Without IP	38	8	46

Total		90	15	105

**Table 3:** Statistics of the inspection results of the observation group.

Observation group (*n* = 105 cases)		PET		Total
		With IP	Without IP	
DWI after denoising	With IP	79	3	82
	Without IP	8	13	21

Total		89	16	105

**Table 4:** Comparison of average ADC values between the focal core and the IP region (×10^−3 ^mm^3^/s).

	Cerebral infarction lesion center	IP region
Control group (*n* = 59 cases)	0.342 ± 0.106	0.803 ± 0.149
Observation group (*n* = 82 cases)	0.336 ± 0.102	0.793 ± 0.136

## Data Availability

The data used to support the findings of this study are available from the corresponding author upon request.
